# Microstructure Evolution of Ti-45Al-8.5Nb-0.2W-0.2B-0.02Y Alloy during Long-Term Thermal Exposure

**DOI:** 10.3390/ma13071638

**Published:** 2020-04-02

**Authors:** Zhiyuan Chen, Zhengkun Cai, Xiaosong Jiang, Song Chen, Zewen Huang, Hongliang Sun

**Affiliations:** 1 Key Laboratory of Advanced Technologies of Materials, Ministry of Education, School of Materials Science and Engineering, Southwest Jiaotong University, Chengdu 610031, China; zhiyuan_c19@163.com (Z.C.); caizkcd@163.com (Z.C.); xsjiang@home.swjtu.edu.cn (X.J.); schen2012@home.swjtu.edu.cn (S.C.); 2 School of Metallurgy and Materials, The University of Birmingham, Birmingham B15 2TT, UK; zewenhuang@swjtu.edu.cn

**Keywords:** titanium aluminides, microstructure, phase transformations, thermal stability, electron microscopy

## Abstract

The hot-rolled alloy Ti-45Al-8.5Nb-0.2W-0.2B-0.02Y was exposed to 700 °C air for up to 10,000 h. The changes in microstructure were observed using scanning and transmission electron microscopies. It was found that the α_2_ laths, α_2_ + γ lamellae, and B2(ω) structure of the alloy showed thermodynamic instability. There were three types of phase transformation in the alloy during long-term thermal exposure. The first was α_2_ → γ, which occurs in the interior and boundary of the α_2_ + γ lamellae. The second was α_2_ + γ → B2(ω), which occurs on the α_2_ + γ boundary. In addition, B2(ω) also precipitates on the γ/γ interfaces. The third was B2(ω) → γ, which describes the precipitation of micron-scale γ phases in the B2(ω) area after thermal exposure of 5000 h. The volume fraction and size of the B2(ω) area and equiaxed γ grains continued to increase throughout the exposure process. Large-sized γ grains and a B2 area of tens of microns appeared in the microstructure after long-term thermal exposure. The volume fractions of the B2 area and the equiaxed γ grains after thermal exposure of 10,000 h reached 16.8% and 63.2%, respectively.

## 1. Introduction

γ-TiAl-based intermetallic alloys have been widely used in aero-engine and automobile applications to replace Ni-based and Ti-based alloys and steels [1,2,3,4]. Compared to traditional TiAl alloys, high-Nb-containing TiAl alloys have higher tensile strength, better oxidation resistance, and high-temperature creep resistance, which have received high attention and in-depth research from various circles [5,6,7,8,9,10]. The high-Nb-containing TiAl alloys are typically used at high temperatures ranging from 600 °C to 900 °C. The basic characteristics of TiAl-based alloys require long-term thermal stability in the presence of air. Microstructure and mechanical properties are maintained throughout the service life of the components. Therefore, it is very important to study the long-term thermal stability of high-Nb-containing TiAl alloys under high-temperature conditions.

The refractory metal Nb is an extremely effective alloy element that improves the high-temperature strength and high-temperature oxidation resistance of the TiAl alloy. Nb is an effective beta phase stabilizer in TiAl-based alloys [11,12]. Adding Nb can significantly refine the microstructure of TiAl alloy and improve the yield strength [5,13,14]. In addition, the addition of Nb at a high content reduces the stacking fault energy and increases the diffusion activation energy of the TiAl alloy, enhancing the plasticity, creep resistance, and other high-temperature stabilities of the material [5,15,16]. However, segregation problems are prone to occur during the casting process due to the significant partitioning effects of the refractory metal Nb. After cooling to room temperature, an ordered B2(ω) structure, α_2_ lamellae, and equiaxed γ grains will form [6,17,18,19,20]. γ, α_2_, and B2 are common phases in high-Nb-TiAl alloys. The γ phase is an ordered face-centered-cubic structure (fcc), and the atomic ratio of Ti to Al in the nominal composition is 1:1 [21]. The atomic ratio of Ti to Al under the nominal composition of the α_2_ phase is 3:1, which is an ordered hexagonal α phase [22]. In high-Nb-TiAl alloys, Nb will replace Ti or Al atoms when it is a solid solution in the γ phase, forming Nb/Al reaction atom defects, and Nb will also replace Ti atoms when it is a solid solution in the α_2_ phase [23,24]. Cooling the high-temperature β-phase to room temperature will transform it into an ordered β-phase, commonly referred to as the B2 phase. The B2 phase is a body-centered-cubic structure (bcc), and the atomic ratio of Ti to Al is 1:1 under the nominal composition [25]. Previous studies have shown that when TiAl-based alloys are used at high temperatures for a long time, their microstructure is prone to change. This will affect the mechanical properties [26,27].

Hu et al. [28] studied the microstructure stability of the full lamellar Ti-48Al-2Cr-2Nb-1B alloy after thermal exposure at 700 °C for 3000 h. It was found that the α_2_ phase in the alloy almost completely disappeared after exposure for 3000 h. The decomposition of the α_2_ phase led to the decrease in the tensile strength of the alloy. Beschliesser et al. [29] studied the full lamellar Ti–46.5Al-4(Cr, Nb, Ta)-B alloy after thermal exposure for 3500 h, and a part of the coarse α_2_ laths decomposed into fine α_2_ + γ lamellae, and equiaxed γ grains grew during thermal exposure. Huang et al. [30,31,32] found that the long-term thermal stability of high-Nb-TiAl alloys in high-temperature environments (such as 700 °C) is not ideal. The α_2_ laths in the ingot-cast and forged full lamellae will cause extensive phase transformation of α_2_ → γ and α_2_ → B2 during thermal exposure. The equiaxed B2 crystal block (nano or sub-micron size) will also appear on the α_2_ + γ lamellae. This is because the phase transformation of α_2_ + γ → B2 occurs on the α_2_ + γ lamellae. There is always the precipitation of the ω phase in this newly formed B2 phase, thus forming a unique symbiosis phenomenon of the ordered B2 + ω phase. The formation and structure of the ω phase is relatively complicated, which has attracted the attention of researchers, and it is generally considered an ordered hexagonal phase [33,34,35]. Previous studies have shown that there are some differences in the long-term thermal stability of TiAl alloys due to different alloy elements and manufacturing methods [27,28,29,30,31,32,36]. Therefore, it is necessary to study the microstructure changes of different TiAl-based alloys in detail.

Ti-45Al-8.5Nb-0.2W-0.2B-0.02Y alloy is a high-performance lightweight TiAl alloy developed in recent years with good comprehensive properties [8,37,38,39]. The alloy obtained in the hot-rolled state is a duplex microstructure (DP). Most of the thermal stability studies of TiAl-based alloys have focused on the fully lamellar or near-lamellar TiAl alloys, but few have focused on the newly developed duplex TiAl alloy. Therefore, the long-term thermal stability of Ti-45Al-8.5Nb-0.2W-0.2B-0.02Y alloy is studied in detail. This alloy was exposed to air at 700 °C for up to 10,000 h. The aim of this work was to comprehensively characterize and understand the microstructural and phase changes during long-term thermal exposure by scanning electron microscopy (SEM) and transmission electron microscopy (TEM).

## 2. Materials and Methods

The alloy Ti-45Al-8.5Nb-(W, B, Y) (Ti-45Al-8.5Nb-0.2W-0.2B-0.02Y) (at.%) was chosen for the present study. The original material was provided at the State Key Laboratory of New Metal Materials, University of Science and Technology Beijing (Beijing, China). It was taken from a plasma-melted ingot and then subjected to hot rolling three times. The hot-rolled blank was air-cooled to room temperature to ensure a duplex microstructure (DP). The sample was taken from the alloy ingot by electro-discharge machining, placed in an air circulation furnace, and then subjected to thermal exposure at 700 °C in an air-circulating furnace for the durations of 3000, 5000, and 10,000 h, respectively. Thermocouples were used to monitor the temperature throughout this process, using an BR-14MT thermocouples provided by Brother Corp., Zhengzhou, Henan, China. The samples used in the experiment were all taken from the core of the Ti-45Al-8.5Nb-(W, B, Y) alloy after thermal exposure, and were processed by DK7740 wire electrical discharge machining (WEDM) (Jiangzhou CNC, Taizhou, Jiangsu, China).

The microstructure of the alloy before and after thermal exposure was observed by backscattered electron microscopy, using an FEI Quanta FEG 250 scanning electron microscope provided by FEI company, Hillsboro, OR, USA, operating at 20 kV. Transmission electron microscopy investigation was carried out on an FEI Tecnai G2 F30 microscope operating (FEI company, Hillsboro, OR, USA) at 200 kV. The TEM samples were completed by ion thinning. TEM-EDX was conducted using an Octane SDD analytical EDX micro analyzer (FEI company, Hillsboro, OR, USA). The EDX analysis results listed in this paper are the average of eight locations for each microstructural feature.

Image analysis was conducted on SEM backscattered electron (BSE) micrographs to measure the size and volume fraction of different phases and structures before and after the thermal exposure stages. Measurements were performed on at least 15 randomly selected photomicrographs using the average linear intercept method. For all measured components and geometric parameters, the mean is expressed as the standard deviation and represents the uncertainty of statistical analysis.

## 3. Results

### 3.1. BSE Images of Unexposed and Exposed Microstructures

**Figure 1:**
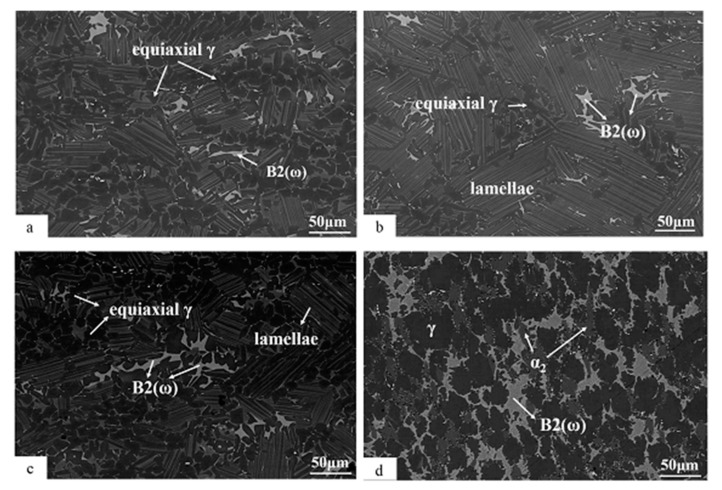
Back-scattered SEM (scanning electron microscopy) images of Ti-45Al-8.5Nb-(W,B,Y) alloy after different exposure times at 700 °C: (**a**) 0, (**b**) 3000, (**c**) 5000, and (**d**) 10,000 h.

Figure 1a shows the backscattered electron (BSE) image of the Ti-45Al-8.5Nb-(W, B, Y) alloy before thermal exposure. This alloy had a duplex microstructure (DP) after hot rolling and cooling to room temperature. It consisted of a large number of equiaxed γ grains and α_2_ + γ lamellae, with a small amount of B2(ω) structure. The B2(ω) structure is bright white in the backscattered image because it was rich in heavy metal elements. The B2(ω) structure in this alloy appeared around the α_2_ + γ colony boundaries, not inside the α_2_ + γ lamellae. In addition, the larger B2(ω) phase also appeared in the equiaxed γ grain area and connected with the fine α_2_ laths. The B2 phase is generally considered as the ordered phase of the high-temperature β phase at room temperature because of the coring effects of the high-alloyed TiAl alloy during solidification [40]. The α_2_ + γ lamellae has a certain degree of bending due to the high-temperature hot rolling, and its distribution has no certain directionality. Careful observation shows that there is a small amount of boride in the microstructure of the alloy, which are scattered in the entire microstructure.

Figure 1b–d shows the BSE images of the Ti-45Al-8.5Nb-(W, B, Y) alloy during the thermal exposure to 10,000 h. It can be seen from Figure 1 that the equiaxed γ grains, B2(ω) structure, and α_2_ + γ lamellae in the alloy showed thermal instability after thermal exposure. After long-term thermal exposure, B2(ω) and equiaxed γ grains increased, while α_2_ + γ lamellae gradually disappeared. Quantitative statistics of the phase size and volume fraction of the alloy after different exposure times at 700 °C are shown in Table 1. The volume fraction of B2(ω) increased slowly during 0–5000 h of thermal exposure, but increased rapidly after exposure of 10,000 h. The volume fraction of the B2(ω) structure was about 16.8% after 10,000 h of thermal exposure, which was 6.6 times higher than that before thermal exposure. The average size of the B2(ω) structure was about 28.8 μm, which is 1.79 times more than before the thermal exposure. Similar to the B2(ω) structure, the volume fraction and size of the equiaxed γ grains increased with the increase in thermal exposure. The average size of equiaxed γ grains grew significantly after 10,000 h of exposure, reaching about 47.5 μm, which increased by 1.27 times compared to that before exposure. The large-area B2(ω) phase and large-size equiaxed γ grains generated during the long-term thermal exposure of high-Nb-containing TiAl alloys were observed for the first time. This may be the phenomenon of phase transformations α_2_ → γ, α_2_ → B2(ω), or α_2_ + γ → B2(ω) that have been extensively reported in the study of long-term thermal stability of high-Nb-containing TiAl alloys [30,31,32,41]. This is consistent with the gradual decrease in the α_2_ + γ lamellae during long-term thermal exposure, as shown in Figure 1 and Table 1. The volume fraction and size of α_2_ + γ lamellae decreased by 1/2 and 26.5%, respectively, after thermal exposure for 10,000 h.

In addition, it should be noted that acicular phases began to precipitate in B2(ω) after the alloy was exposed to 5000 h. This acicular phase will continue to grow with the increase in exposure time. After thermal exposure of 10,000 h, the size of the acicular phases in the B2(ω) area reached the micron level, as shown in Figure 1d. The acicular phase precipitated in the B2(ω) area will be discussed in detail by TEM.

### 3.2. TEM Examination of Unexposed Microstructures

**Figure 2:**
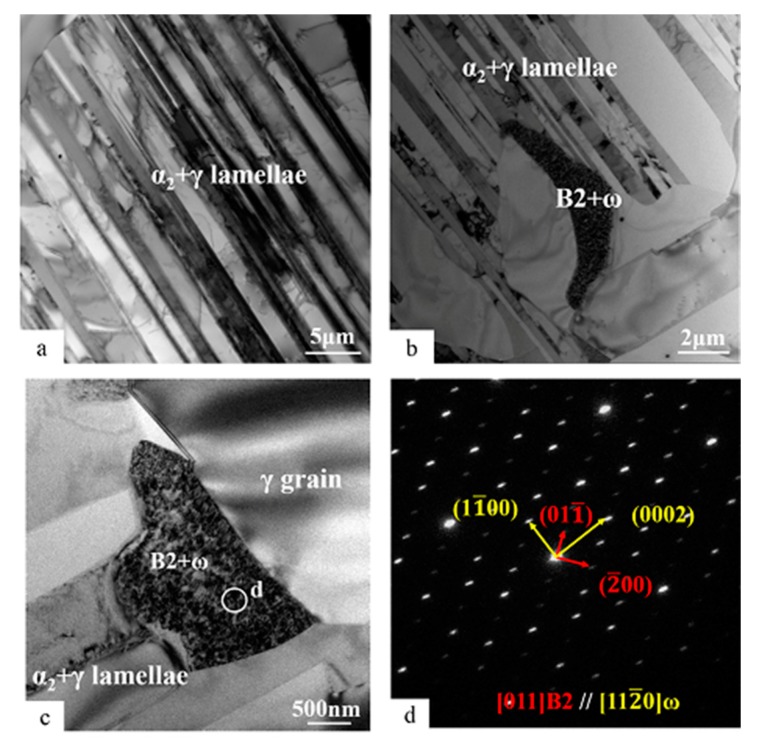
Transmission electron microscopy (TEM) bright-field (BF) images of unexposed structures in Ti-45Al-8.5Nb-(W,B,Y) alloy: (**a**) α_2_ + γ lamellae, (**b**,**c**) B2(ω) area, and (**d**) the selected area diffraction pattern (SADP) taken from B2(ω) section indicated in (**c**).

TEM bright-field (BF) images of the Ti-45Al-8.5Nb-(W, B, Y) alloy before thermal exposure are shown in Figure 2. As can be seen from Figure 2a, α_2_ + γ lamellae comprise α_2_ (dark) and γ lamellae (bright) alternately arranged, and their thickness is not uniform. The α_2_ lamellae usually comprise several thinner α_2_ lamellae, of which very thin γ lamellae form α_2_ + γ lamellar packets. There are many small α_2_ + γ lamellae appearing at the positions of the original α_2_ laths in the alloy. The direction of the α_2_ + γ lamellae is almost parallel to the direction of the original α_2_ laths, and the lamellar interface is basically coherent. According to the relevant literature reports, the α_2_ phase and γ lamellae in lamellar colonies satisfy a certain orientation relationship in crystallography: {111}γ // {0001}α_2_ and <1$\bar{1}0$>γ // <11$\bar{2}$0>α_2_ [32,42]. These crystallographic orientations are stable at different stages of thermal exposure. It can be seen from Figure 1a,b and Table 1 that the α_2_ laths of the Ti-45Al-8.5Nb-(W, B, Y) alloy after plasma melting, hot rolling three times, and air cooling to room temperature will exist in α_2_ + γ lamellae with the metastable structure. As the duration of thermal exposure increases, the volume fraction of the metastable α_2_ laths will decrease. These metastable α_2_ laths are an important source of instability of the alloy. In the process of thermal exposure, the transformation of α_2_ → γ is likely to occur in order to balance the structure and composition of the alloy [30].

Figure 2b shows a typical location of B2(ω) before thermal exposure, which is located on the boundary of α_2_ + γ lamellae. Part of B2(ω) is connected with α_2_ laths, and the rest of B2(ω) is surrounded by equiaxed γ grains, where the ω grains are fully integrated into the B2 area, as shown in Figure 2c. Selected diffraction analysis in the B2(ω) area of Figure 2c is shown in Figure 2d. Due to the astigmatism when photographing with the electron microscope, the selected-area diffraction spots in Figure 2d are elongated, but this has no effect on the experimental results. The typical orientation relationship between the B2 phase and the ω phase in the direction of [011]B2 is obtained: [011]B2 // [11$\bar{2}$0]ω. It is further confirmed that there is a symbiotic relationship between B2 and ω phases in the microstructure of the hot-rolled Ti-45Al-8.5Nb-(W, B, Y) alloy at room temperature like other TiAl-based alloys. It is also shown that there are only B2 and ω phases in the B2 area. In addition, there is another orientation relationship between the B2 phase and ω phase in TiAl-based alloys: <1$\bar{1}$1>B2 // <0001>ω [33,43].

The composition of equiaxed γ grains, α_2_ + γ lamellae, and the B2(ω) structure in the Ti-45Al-8.5Nb-(W, B, Y) alloy before thermal exposure was analyzed by TEM-EDS, and the corresponding atomic content percentage was obtained, as shown in Table 2. This table also lists the partition factor k of the alloying elements Ti, Al, Nb, and W. We can see that the B2(ω) phase is richer in the heavy metal elements Nb and W than the γ and α_2_ phases, but the Al content is relatively low. Nb and W show similar distribution laws. The partition factor k(β/γ) and k(β/α_2_) of W is 3.00, which is higher than the corresponding element Nb. However, the content of W is relatively low. From the backscattering results in Figure 1, W does not hinder the large-scale phase transformation. On the contrary, as the W content in the γ and α_2_ phases is substantially the same, it may provide internal conditions for a large number of α_2_ → γ transformations. In addition, the partition factor k of elements other than W is very close to the equilibrium value of 1. This fully shows that the different phases have similar chemical compositions in the alloy. From the perspective of element diffusion, the composition thresholds for phase transformations α_2_ → γ and α_2_ → B2(ω) are low. This is similar to the phenomenon observed in the reported TiAl alloys (Ti-44Al-8Nb-1B) with high Nb content. Similar chemical compositions can provide the possibility for a large number of phase transformations [37,41].

### 3.3. Observation of Microstructure Changes during 10,000 h Exposure by Transmission Electron Microscopy

#### 3.3.1. Bulk and Flaky Large γ Grains Appear after 5000 h and Exposure

**Figure 3:**
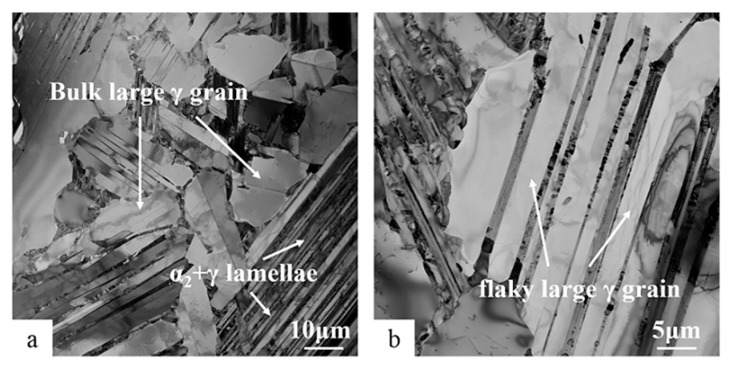
TEM BF images of γ grains in Ti-45Al-8.5Nb-(W, B, Y) alloy after long-term exposure, showing the transformation of (**a**) bulk large γ grains after 5000 h exposure and (**b**) flaky large γ grains after 10,000 h exposure.

The microstructure of the Ti-45Al-8.5Nb-(W, B, Y) alloy was observed in detail by transmission electron microscopy during long-term atmospheric exposure at 700 °C. For the first time, large-sized γ grains were observed in the alloy. Figure 3a shows that bulk γ grains appeared in the structure after the alloy was exposed to 5000 h. The size of these γ grains reached tens of microns, which is consistent with the data results in Table 1. From Figure 3a, it can also be found that these massive γ grains are distributed on the α_2_ + γ lamellae boundary or parallel to the α_2_ + γ lamellae. At this time, the size of α_2_ laths and γ lamellae inside α_2_ + γ lamellae is not very different. However, flaky large γ grains grew in α_2_ + γ lamellae after the alloy was exposed to 10,000 h, as shown in Figure 3b. The flaky γ grains were significantly thicker than the fine α_2_ laths. The results show that the γ grains (including γ in α_2_ + γ lamellae) of the alloy grew continuously in thermal exposure from 5000 to 10,000 h, which is also consistent with the results of backscatter in Figure 1d.

**Figure 4:**
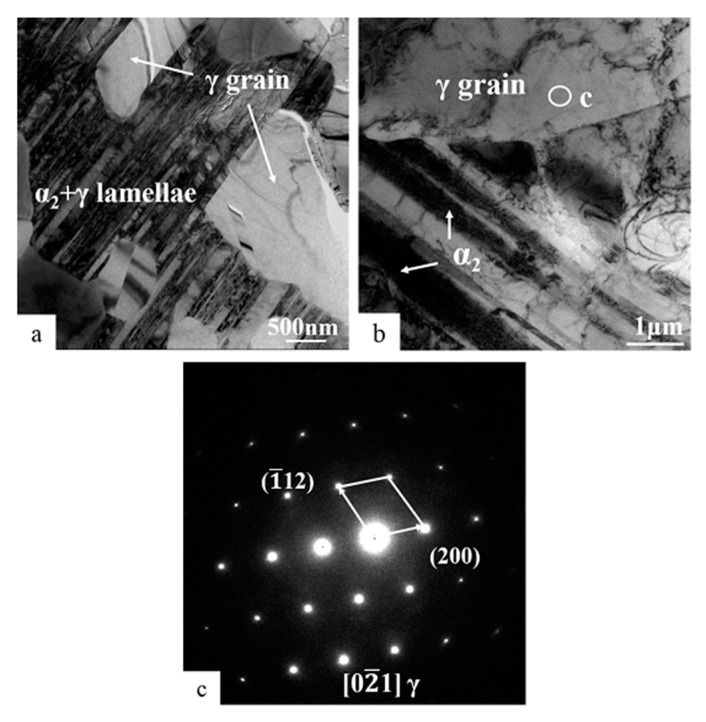
TEM BF images from Ti-45Al-8.5Nb-(W, B, Y) alloy showing the distribution of the γ grains with increasing exposure: (**a**) 5000 and (**b**) 10,000 h, and (**c**) the SADP from the γ matrix transformed by α_2_ decomposition in (**b**).

In order to analyze the growth cause of large-sized γ grains in Ti-45Al-8.5Nb-(W, B, Y) alloy during long-term exposure at 700 °C, γ grains in the alloy after exposure of 5000 and 10,000 h were observed and analyzed with TEM, as shown in Figure 4. It can be seen from Figure 3b and Figure 4a that there are massive γ grains inside and outside α_2_ + γ lamellae, which may be due to the large amount of α_2_ → γ decomposition in α_2_ + γ lamellae. The γ grains formed in α_2_ + γ lamellae originate from the parallel decomposition of coarse α_2_ laths. These decompositions typically occur at coarse α_2_ laths or α_2_ aggregation. The results show that the microstructure of the coarse α_2_ laths and aggregated α_2_ laths show thermodynamic instability. Huang et al. [30,31] discussed the decomposition mechanism in more detail. Unlike other TiAl-based alloys, the parallel decomposition of α_2_ laths persists in the entire thermal exposure process, even when thermally exposed to 10,000 h. The γ lamellae in α_2_ + γ lamellae will gradually become spherical. However, the fine α_2_ laths always exist, showing good thermal stability. Generally, the large-sized γ grains around α_2_ + γ lamellae gradually decompose from α_2_ laths in the adjacent α_2_ + γ lamellae, and spheroidization will take place in itself.

After thermal exposure of the Ti-45Al-8.5Nb-(W, B, Y) alloy for 10,000 h, it was found that the formed large-sized γ grains had surrounded the residual α_2_ laths, as shown in Figure 4b. The presence of fine α_2_ laths was still observed at this time. Figure 4c is the selected diffraction of the γ grain area in Figure 4b, showing the orientation relationship of [0$\bar{2}1$]γ. This further confirms the instability of α_2_ laths during thermal exposure. The α_2_ structure will dissolve into the γ phase in a large area, which is α_2_ → γ decomposition. This shows that the spherical γ grains continue to grow, and together with the γ matrix in the original structure, form a broader new γ matrix with irregular boundaries. The internal and external thermodynamic instability of α_2_ + γ lamellae will promote the rapid increase in size and quantity of γ grains in the alloy after long-term thermal exposure.

#### 3.3.2. A Large Amount of B2(ω) is Precipitated at the α_2_ + γ Lamellae Boundary and the γ/γ Interfaces during Long-Term Exposure

**Figure 5:**
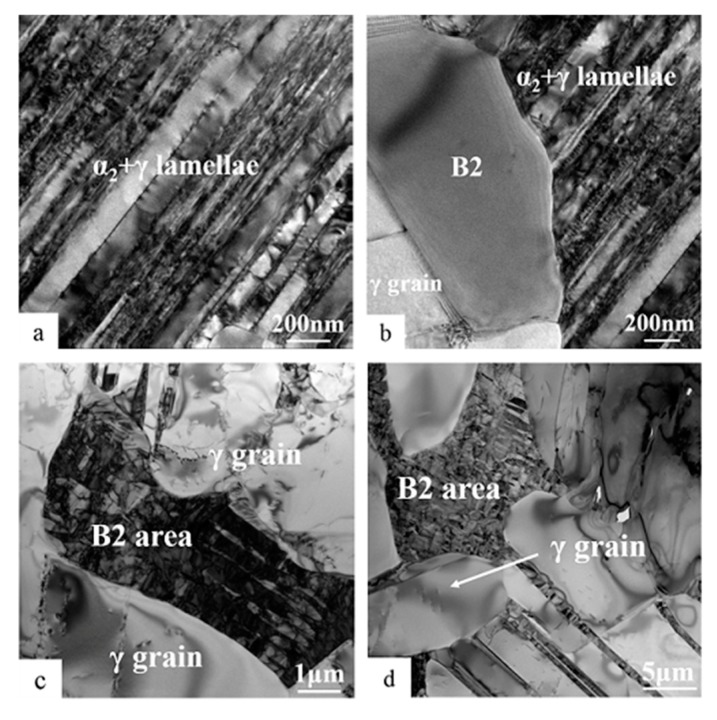
TEM BF images after 5000 h exposure showing (**a**) the microstructures at α_2_ + γ lamellae and (**b**) the precipitated B2 phase at lamellae boundaries; (**c**,**d**) TEM BF images after 10,000 h exposure showing the precipitated B2 phase at γ/γ interfaces.

From the BSE results of Figure 1 and Table 1, it is known that a large number of B2(ω) structures were grown in the Ti-45Al-8.5Nb-(W, B, Y) alloy after long-term thermal exposure. The bright-field (BF) images in Figure 5a,b are the internal and boundary morphologies of α_2_ + γ lamellae after thermal exposure of the alloy for 5000 h, respectively. The Ti-45Al-8.5Nb-(W, B, Y) alloy precipitates large-sized B2 phases at the α_2_ + γ lamellae boundary after 5000 h of exposure. This indicates that not only γ grains but also the B2(ω) structure may be formed at the boundary of α_2_ + γ lamellae. The growth of B2(ω) at the boundary of α_2_ + γ lamellae may be due to the phase transformation of α_2_ + γ → B2(ω). Research on the thermal stability of TiAl-based alloys has reported that α_2_ laths inside α_2_ + γ lamellae are vertically decomposed into the B2(ω) structure [27,32,41,42]. However, there is no large-scale B2 precipitated in α_2_ + γ lamellae during long-term thermal exposure. This shows that the phenomenon of direct decomposition into B2(ω) within the α_2_ laths has little or no existence, but only the parallel decomposition into γ grains discussed previously. During the long-term thermal exposure, some α_2_ + γ lamellae will have a tendency to transition to the B2 phase, which will affect the adjacent γ tissue [30]. The present study further supports this view, indicating that the B2 structure is more thermodynamically stable than the α_2_ + γ lamellae at 700 °C. However, this study found that the transformation can occur in the Ti-45Al-8.5Nb-(W, B, Y) alloy in the form of tens of microns. This will result in the entire lamellae bring transformed into B2 grains, showing a strong phase transformation drive. The internal reason is not clear, which may be related to the composition segregation of the alloy.

After the Ti-45Al-8.5Nb-(W, B, Y) alloy was exposed to 5000 and 10,000 h, it was found that the B2 area was surrounded by multiple large γ grains, as shown in Figure 5c,d. This B2 area similar to the γ/γ interfaces may be the supersaturation of Nb in the precipitated γ grains [44,45]. Due to the supersaturation of Nb in the matrix (α_2_ and γ), the thermodynamic instability of the alloy may lead to the precipitation of the B2 area. It will reduce the free energy of the alloy. Comparing Figure 5c,d, it can be found that the B2 area continued to increase during the thermal exposure from 5000 to 10,000 h. This indicates that the alloy continued to generate the new B2 phase during long-term thermal exposure. The boundaries of α_2_ + γ lamellae and γ/γ interfaces continuously transformed to generate new B2 area, leading to the large B2 area observed in the alloy after exposure of 10,000 h.

In addition, it can be noticed that the B2 area after thermal exposure of 5000 and 10,000 h is different from the B2 area without exposure (Figure 2c). There have been some new ordered phases generated in the B2 area, showing an obvious strip shape. At this time, the phase composition in the B2 area is not just a simple B2(ω) symbiotic structure. It can be seen that the internal structure of the B2 area is not stable under long-term thermal exposure. As the exposure time continues to increase, the internal structure of the B2 area also changes simultaneously, which will be analyzed and discussed separately below.

#### 3.3.3. The γ Phase Precipitated in the B2 Area after 5000 and 10,000 h Exposure

**Figure 6:**
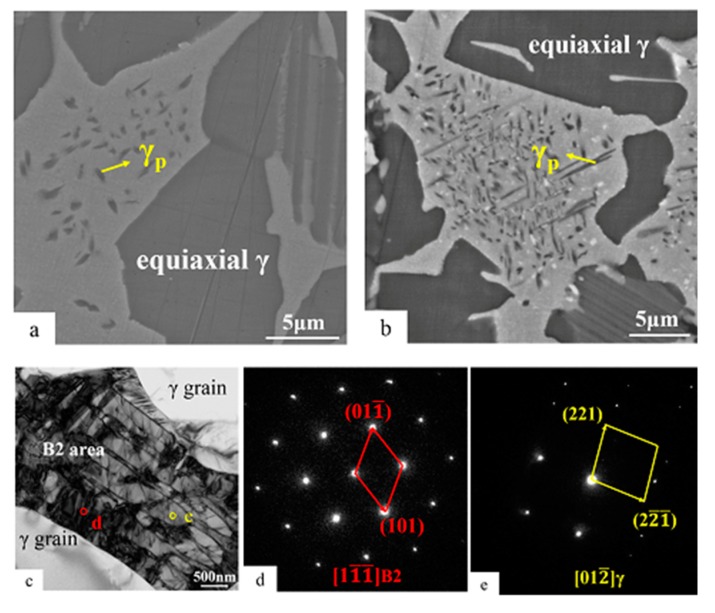
Backscattered electron (BSE) images of B2 area structures in Ti-45Al-8.5Nb-(W, B, Y) alloy after (**a**) 5000 and (**b**) 10,000 h exposure; (**c**) TEM BF images of B2 area after 5000 h exposure and (**d**,**e**) the SADPs taken from B2 and the precipitated γ phase in (**c**), respectively.

When the Ti-45Al-8.5Nb-(W, B, Y) alloy was exposed at 700 °C for more than 5000 h, the B2(ω) area showed obvious structural instability. Figure 6a,b are amplified BSE images of the B2 area after thermal exposure of 5000 and 10,000 h, respectively. It can be found that a black acicular phase precipitated in the B2 area, and reached the micron level after 5000 h of exposure. Moreover, the size of the acicular phase further increased after the exposure of 10,000 h, and the quantity also significantly increased. These black acicular phases are scattered and disordered in the B2 area. In previous related studies, the B2 area has been considered to be thermally stable [30,36,42]. In fact, this conclusion may only hold after short-term thermal exposure, while phase transformation will occur in the B2 area after long-term thermal exposure of the duplex Ti-45Al-8.5Nb-(W, B, Y) alloy. It is worth noting that a white punctate phase precipitated in the B2 area after thermal exposure for 10,000 h, as shown in Figure 6b. This phenomenon was not found in the BSE images after 5000 h exposure. The white punctate phase may be the ω phase coexisting with B2 in the early stage, and this judgment needs to be studied in the next stage. This paper focuses on the large-scale acicular phases precipitated in the B2 area.

Figure 6c is a transmission bright-field image of the B2 area after thermal exposure for 5000 h. It can be seen that some long strip structures are clearly precipitated on the B2 matrix. Selective diffraction analysis of the B2 matrix and the precipitated strip structure are shown in Figure 6d,e, respectively. Figure 6d shows the orientation of [1$\bar{1}\bar{1}$]B2 and Figure 6e shows the orientation of [01$\bar{2}$]γ, indicating that the new stripe phase precipitated in the B2 area (the black acicular phase under the backscattered electron image) is the γ phase. This is a significant difference in the thermal stability between the Ti-45Al-8.5Nb-(W, B, Y) alloy and other TiAl-based alloys. The acicular γ phase in the B2 area is rarely found in the literature on thermal stability of TiAl-based alloys, but it has been reported in other cases [46,47]. Song et al. [41] found that the γ phase precipitated in the B2(ω) area after long-term annealing at 850 °C for Ti-45Al-8.5Nb-0.2B alloys and reported the orientation relationship between the precipitated γ phase and ω grain. They think that the γ phase in the B2(ω) area is the result of growth from the ω phase variant. The Nb content at the boundary of the growing ω variant can be lower than the Nb content inside the ω grains, while the Al content follows the opposite trend. The decrease in Nb and increase in Al lead to the formation of the γ phase at the boundary of the growing ω grains. In addition, the formation mechanism of the γ phase may also be attributed to the direct transformation from the B2 phase to γ phase during long-term thermal exposure. In other words, precipitation of the γ phase in the Ti-45Al-8.5Nb-(W, B, Y) alloy during long-term thermal exposure can be described as the transformation B2(ω) → γ.

## 4. Discussion

The hot-rolled duplex Ti-45Al-8.5Nb-(W, B, Y) alloy is exposed to air at 700 °C for up to 10,000 h. The experimental results show that the microstructure of the duplex TiAl-based alloy will be significantly affected by long-term thermal exposure. It was clearly revealed by SEM and TEM that the structures of α_2_ laths, α_2_ + γ lamellae, and B2(ω) in the alloy presented thermodynamic instability. These microstructures have undergone extensive microstructural decomposition, indicating that the hot-rolled Ti-45Al-8.5Nb-(W, B, Y) alloy is in the non-equilibrium state. This kind of non-equilibrium state is mainly caused by a large number of α_2_ lamellae with irregular distribution. It can be known from Figure 1 and Table 1 that the volume fraction of α_2_ lamellae decreases due to the instability of the microstructure after long-term exposure. A large amount of α_2_ laths dissolved continuously during exposure, which made the alloy structure return to the stable state. This phenomenon may be due to the rate of alloy cooling to room temperature and the coring effect during solidification [41]. The cooling rate may cause the structure to be in non-thermodynamic equilibrium at room temperature. In addition, the distribution of elements in the alloy will be uneven during solidification. In places rich in Ti and Nb, more and rougher α_2_ lamellae formed. In places rich in Al, the α_2_ lamellae formed were small and fine. These heterogeneous distributed α_2_ lamellae are the source of phase transformation during long-term thermal exposure. As a result, the alloy has three typical microstructural changes during long-term thermal exposure:

The first is that the volume fraction of equiaxed γ grains increases continuously with the thermal exposure. In the process of exposure from 5000 to 10,000 h, the size of γ grains increased rapidly (see Table 1). Large-sized and strip-shaped γ grains were observed under transmission electron microscopy after long-term exposure (Figure 3). These large γ grains were distributed in the interior and boundary of α_2_ + γ lamellae. Huang et al. [30,31,32] reported the vertical and parallel decomposition of α_2_ + γ lamellae during long-term thermal exposure. The B2(ω) structures are the product of vertical decomposition inside α_2_ + γ lamellae of full-layer or near-layer TiAl-based alloys. However, a large number of B2(ω) structures were not found inside α_2_ + γ lamellae after thermal exposure of the hot-rolled Ti-45Al-8.5Nb-0.2W-0.2B-0.02Y alloy, but the equiaxed γ grains appeared (Figure 4a). At the same time, γ grains also formed on the α_2_ + γ lamellae boundary. The γ grains formed at these two different locations transformed from α_2_ lamellae. It is clear that α_2_ → γ exists widely in the entire process of thermal exposure. Table 2 indicates that the partition factor (k) of each element of α_2_ and γ is close to 1, and the transformation of metastable phase α_2_ into γ grains easily occurs. These transformed γ grains will gradually spheroidize with the thermal exposure and then merge in the γ matrix. These transformations usually occur in coarse α_2_ laths or α_2_ aggregates, which is to achieve an optimal balance by reducing the content of the metastable phase α_2_.

The second is that a large amount of B2(ω) structure is precipitated in the alloy during the exposure. According to Table 1, B2(ω) grows slowly before 3000 h of thermal exposure. This may be caused by the smaller diffusion coefficients of Ti, Al, Nb, and other elements in B2(ω), α_2_, and γ [48]. Unlike the reported thermal stability of other high-Nb TiAl-based alloys, the large-scale B2(ω) structure that appeared after thermal exposure at 700 °C for 10,000 h was the first discovery. Generally, there are few B2 structures in the initial state of the Y-containing TiAl alloys, which is the biggest advantage of the developed Y-containing TiAl alloys [8,37,38]. Therefore, the appearance of a large number of B2(ω) structures after long-term thermal exposure should be given enough attention. These large amounts of B2(ω) appear on the α_2_ + γ lamellae boundary, and there exists α_2_ + γ → B2(ω). These B2(ω) are formed by consuming α_2_ and γ lamellae. The phenomenon of the transformation α_2_→B2(ω) has not been discovered inside the α_2_ + γ lamellae, which needs further study. From the distribution behavior of alloy elements among phases, there is not much difference between the B2(ω) structure and α_2_ + γ lamellae [42]. This is also confirmed by Table 2. Therefore, the transformation from α_2_ + γ lamellae to B2(ω) does not require a large number of diffusions of major elements. B2(ω) has a similar chemical composition to α_2_ + γ lamellae, which provides the driving force for element diffusion for α_2_ + γ → B2 (ω). Another reason why B2(ω) appears on the boundary of α_2_ + γ lamellae is due to the assistance of the defects of α_2_ + γ interfaces and the micro-segregation of alloy elements at the interface. These will promote the nucleation of B2(ω) [49,50]. In addition, when the thermal exposure reached 10,000 h, the B2 area began to precipitate at the γ/γ interfaces. This may be caused by the supersaturation of heavy metal Nb, and the specific internal cause may be considered from thermodynamics and dynamics [46]. The alloy had a symbiotic structure of B2 and ω before thermal exposure, but an independent B2 phase was found after 5000 h of thermal exposure (Figure 5b). Therefore, whether B2 and ω are still symbiotic after long-term thermal exposure is worthy of further discussion.

The third is that the duplex Ti-45Al-8.5Nb-(W, B, Y) alloy precipitates micron-sized γ grains in the B2 area after 5000 h thermal exposure (Figure 6). This is the most prominent feature of the alloy’s long-term thermal stability. At present, the internal mechanism of γ phase precipitation in the B2 area of TiAl-based alloys after long-term thermal exposure is not clear. Both the B2 phase and the ω grains in the B2 area (if present after long-term thermal exposure) may be responsible for the precipitation of the γ phase. Here, we can use B2(ω) → γ to represent this transformation phenomenon. Moreover, the γ precipitated in the B2 area should have a certain orientation relationship with the B2 matrix. In this paper, only the precipitated phases in the B2 area are identified. The specific growth mechanism of the γ phase precipitated in the B2 area needs to be further studied.

## 5. Conclusions

(1) The hot-rolled Ti-45Al-8.5Nb-0.2W-0.2B-0.02Y alloy has a duplex microstructure (DP) at room temperature. During thermal exposure to air at 700 °C for 10,000 h, the microstructure of the alloy exhibited thermodynamic instability. The metastable α_2_ phase is an important internal cause for the transformation of the microstructure.

(2) There are a lot of equiaxed γ grains and B2 area in the alloy structure after long-term thermal exposure. The volume fractions of the B2(ω) area and the equiaxed γ grains after thermal exposure for 10,000 h will reach 16.8% and 63.2%, respectively, and their sizes will reach 28.8 and 47.5 μm, respectively.

(3) When the Ti-45Al-8.5Nb-0.2W-0.2B-0.02Y alloy is exposed to air at 700 °C for 5000 h, the micron-sized and acicular γ grains precipitate in the B2 area. These γ grains precipitated in the B2 area will continue to grow with the thermal exposure time. This process can be described as the phase transformation B2(ω) → γ.

(4) The precipitated B2(ω) structure is formed by phase transformation α_2_ + γ → B2(ω), which occurs at the boundary of α_2_ + γ lamellae. No large-sized B2(ω) structure was found inside α_2_ + γ lamellae. The large amount of B2(ω) was mainly attributed to the similar chemical composition with the parent phase. The composition threshold required for α_2_ + γ → B2(ω) transformation is low. In addition, the defects of α_2_ + γ interfaces and the micro-segregation of alloy elements at the interface also promote the nucleation of B2(ω).

(5) The precipitated equiaxed γ grains are formed by decomposition of the phase transformation α_2_ → γ, which occurs at the coarse α_2_ laths or α_2_ aggregates inside and the boundary of α_2_ + γ lamellae. During long-term thermal exposure, these metastable α_2_ tend to reach a new structure balance in the alloy by decomposing into the γ phase.

## Figures and Tables

**Table 1:** The changes in volume fraction and size of the main structures Ti-45Al-8.5Nb-(W, B, Y) after different exposure times at 700 °C.

Exposure Time (h)	Volume Fraction (%)			Size (μm)		
	B2(ω)	Equiaxial γ	α_2_ +γ Lamella	B2(ω)	Equiaxial γ	α_2_ +γ Lamella
0	2.2 ± 0.3	57.6 ± 3.2	40.2 ± 3.5	10.3 ± 1.5	20.9 ± 3.4	36.2 ± 1.3
3000	3.7 ± 0.4	58.5 ± 4.1	37.8 ± 4.2	16.4 ± 3.2	23.3 ± 1.6	45.5 ± 2.7
5000	6.5 ± 0.2	61.3 ± 3.7	32.3 ± 3.8	24.4 ± 2.7	27.2 ± 2.3	32.3 ± 2.4
10,000	16.8 ± 2.4	63.2 ± 4.3	20.0 ± 4.3	28.8 ± 1.1	47.5 ± 3.8	26.6 ± 3.5

**Table 2:** Chemical compositions of the β (B2 + ω), γ, and α_2_ phases in alloy Ti-45Al-8.5Nb-(W, B, Y) before 700 °C exposure. The partition factors for Ti, Al, Nb, and W are included.

Element	Composition of Major Constituents (at. %)			Partition Factor k		
	β (B2 + ω)	γ	α_2_	K(β/γ)	K(β/α_2_)	K(α_2_/γ)
Ti	52.6 ± 1.2	44.5 ± 0.9	52.5 ± 0.8	1.18	1.00	1.17
Al	34.4 ± 0.4	46.0 ± 0.8	37.8 ± 0.7	0.75	0.91	0.82
Nb	12.1 ± 0.2	9.2 ± 0.3	9.4 ± 0.2	1.32	1.29	1.02
W	0.9 ± 0.2	0.3 ± 0.1	0.3 ± 0.1	3.00	3.00	1.00
